# Epigenetic Mechanisms of Learning and Memory: Implications for Aging

**DOI:** 10.3390/ijms21186918

**Published:** 2020-09-21

**Authors:** Samantha D. Creighton, Gilda Stefanelli, Anas Reda, Iva B. Zovkic

**Affiliations:** 1 Department of Psychology, University of Toronto Mississauga, Mississauga, ON L5L 1C6, Canada; samantha.creighton@utoronto.ca (S.D.C.); gilda.stefanelli@utoronto.ca (G.S.); 2 Department of Cell & Systems Biology, University of Toronto, Toronto, ON M5S, Canada; anas.reda@utoronto.ca

**Keywords:** epigenetics, aging, memory, brain

## Abstract

The neuronal epigenome is highly sensitive to external events and its function is vital for producing stable behavioral outcomes, such as the formation of long-lasting memories. The importance of epigenetic regulation in memory is now well established and growing evidence points to altered epigenome function in the aging brain as a contributing factor to age-related memory decline. In this review, we first summarize the typical role of epigenetic factors in memory processing in a healthy young brain, then discuss the aspects of this system that are altered with aging. There is general agreement that many epigenetic marks are modified with aging, but there are still substantial inconsistencies in the precise nature of these changes and their link with memory decline. Here, we discuss the potential source of age-related changes in the epigenome and their implications for therapeutic intervention in age-related cognitive decline.

## 1. Introduction

Memory formation is a core feature of neuroplasticity that allows transient events to produce long-lasting changes in the brain and behavior. The establishment of long-lasting memories requires extensive cellular and molecular changes in brain regions associated with memory formation and maintenance. Some of the best understood molecular markers of memory involve changes in protein synthesis and gene expression [1,2], but their transient nature has initiated a search for more stable molecular markers that can persist for the duration of the memory, particularly epigenetics. Epigenetic mechanisms in general and DNA methylation in particular, have long been studied as self-perpetuating mechanisms for maintaining cellular identity over cycles of cell division and as such, epigenetics was hypothesized to represent a way for our brain to store memories over time [3,4,5,6]. In this review, we describe the evolving understanding of epigenetics in memory by exploring the complementary role these mechanisms play in transient adaptation to changing environmental stimuli, as well as stable changes involved in memory maintenance. Furthermore, we discuss how epigenetic factors are altered with aging and their emerging role as therapeutic targets for age-related cognitive decline.

## 2. A Brief Review of Epigenetic Modifications

### 2.1. Chromatin Structure

DNA is packaged into the nucleus by wrapping in 147 base pair segments around a complex of eight histone proteins from four histone families: two each of histones H2A, H2B, H3, and H4 [7,8]. This histone–DNA complex forms nucleosomes, which serve as building blocks of chromatin that create a physical barrier to transcriptional machinery. The effect of chromatin on transcription is influenced by enzymes that write, read, and erase various epigenetic modifications to either promote or repress transcription, thus conferring a vital regulatory function for gene transcription. Specifically, “writer” enzymes catalyze the addition of epigenetic marks on histone tails or DNA, whereas “eraser” enzymes remove these epigenetic marks. In contrast, “reader” proteins interact with the already established epigenetic marks to initiate downstream effects by recruiting diverse interaction partners, or chromatin remodeling machinery that further regulates chromatin structure and function [9,10].

### 2.2. DNA Methylation and Demethylation

**Figure 1:**
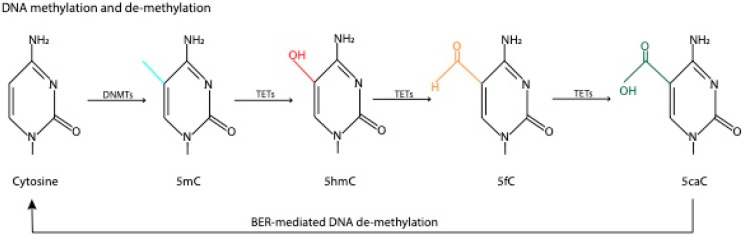
Schematic representation of methylation and de-methylation. Cytosines in the 5′ position are methylated by DNA methyltransferases (DNMTs) to produce 5mC. Ten-eleven translocation (TET) enzymes mediate several oxidation steps to produce 5hmC, 5fC and 5caC, which can undergo base excision repair (BER), in which modified cytosine is replaced by an unmethylated cytosine, resulting in active de-methylation.

DNA methylation is the addition of a methyl group from S-adenosyl methionine (SAM) to the 5′position of a cytosine ring, typically adjacent to a guanine (CpG), to form 5-Methylcytosine (5mC) [11]. However, recent evidence has shown that methylation is not restricted to CpGs and can also occur on cytosines adjacent to other nucleotides, particularly in tissues with low cellular turnover rates, such as the brain [12]. DNA methylation is catalyzed by DNA methyltransferases (DNMTs), enzymes that belong to a conceptual category of epigenetic “writers” that establish or lay down epigenetic marks [13] (Figure 1). Whereas de novo DNMTs (DNMT 3a and 3b) establish new methyl marks, maintenance DNMTs (DNMT1) methylate the complementary DNA strand in dividing cells to perpetuate the mark across cell divisions, thus underlying one form of cellular memory [13]. DNA methylation has been most closely associated with transcriptional repression through the recruitment of chromatin regulators that promote a closed chromatin state, as well as steric interference with transcription factor binding [14,15]. Although some evidence points to a potentially permissive role of 5mC on transcription when it occurs in specific genomic loci [16], it is still predominantly associated with negative transcriptional regulation [17].

Based on evidence that DNA methylation is reversible in the brain [18], there has been extensive effort to identify mechanisms of DNA de-methylation. In 2009, ten-eleven translocation (TET) enzymes were identified as enzymes that bind methylated cytosines (5mC) and catalyze their conversion into hydroxymethylcytosine (5hmC), which can be further deaminated to 5-hydroxymethyluracil and converted back to an unmethylated cytosine via glycolysis-dependent nucleotide excision repair [19,20] (Figure 1). Both TETs and DNMTs are particularly abundant in neurons, where active methylation and de-methylation occurs during learning [21]. Although a majority of early studies focused on 5mC, there is growing evidence that 5hmC is itself an epigenetic mark that may have a positive effect on gene expression and as such, adds complexity to the role of DNA methylation in transcription. For example, 5hmC interacts with the epigenetic “reader” methyl CpG binding protein 2 (MeCP2) to recruit cAMP-response-element-binding protein 1 (CREB1), a crucial transcriptional activator [22]. Additionally, MeCP2 binding at 5hmC-containing DNA within highly expressed genes can facilitate transcription by making chromatin more accessible to transcriptional machinery [23].

### 2.3. Histone Post-Translational Modifications (PTMs)

**Figure 2:**
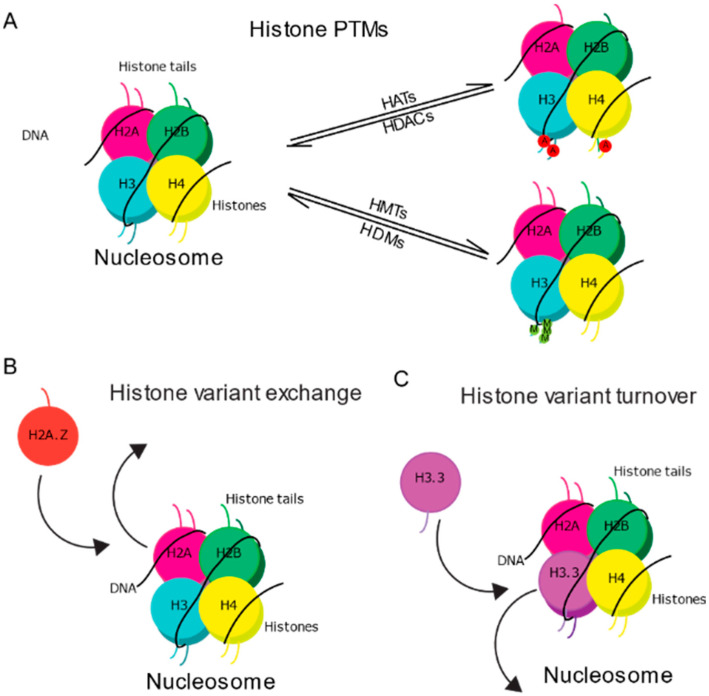
Epigenetic regulation of histones. (**A**) Schematic representation shows organization of DNA around histone octamer. Protruding histone tails can be post-translationally modified by “writer” enzymes, such as histone acetyltransferases (HATs) and histone methyltransferasess (HMTs), and removed by “eraser” enzymes, such as histone deacetylases (HDACs) and histone demethylases (HDMs); Two major mechanisms mediate histone variant functions in chromatin: (**B**) In histone variant exchange, one histone type (H2A in the figure) is removed and replaced with another type (H2A.Z in this example); (**C**) In histone variant turnover, the same histone type is continuously swapping for a new copy of the same variant (figure shows H3.3). Note that histone variant exchange and turnover are not limited to the histone types depicted in the images, as each process can occur with each histone. PTM: post-translational modifications.

Histones are subject to post-translational modifications (PTMs) on amino acid “tails” that protrude from histone proteins and include lysine mono/di/tri methylation and lysine acetylation [7,24,25]. Histone acetylation involves the addition of acetyl groups (-C_2_H_3_O) from acetyl coenzyme A (acetyl CoA) to lysine (K) residues located on the N-terminus of histone protein tails by histone acetyltransferases (HATs) [26], whereas the removal of this mark is regulated by histone deacetylases (HDAC) [27] (Figure 2A). Histone acetylation is typically associated with gene transcription, though the exact mechanism through which acetylation promotes gene transcription is not fully understood. Originally, positive transcriptional outcomes of histone acetylation were attributed predominantly to the weakening of electrostatic interactions between negatively charged DNA and positively charged histones to promote open chromatin states that enable access of transcriptional machinery to associated DNA [27,28]. However, some evidence suggests the neutralization of electrostatic charges between DNA and histones is unlikely [29,30], and that acetylated lysine residues may instead function as marks that are recognized by transcription factors and various reader proteins that signal for further changes in chromatin function and transcription [27,28,31]. Histones can also be modified through methylation, which involves the addition of methyl groups, donated by SAM and catalyzed by histone methyltransferasess (HMTs), to either lysine, arginine, or histidine residues on histone tails [32,33] (Figure 2A). Although histone methylation is thought to be less reversible than other PTMs, a number of histone demethylases have been discovered [34]. In vivo, lysine residues can be mono- di- or trimethylated; arginine residues can be mono- or di-methylated, and histidines can be monomethylated [35,36]. The effects of histone methylation on gene transcription are dependent on the specific residue and the degree (i.e., mono, di, tri) of methylation [36]. For example, H3K4me3 is associated with genes that are poised for activity or are actively transcribed [37,38], whereas H3K27me3 is associated with repressed chromatin, H3K4me with enhancers [39], and H3K4me3 with promoter activity [36]. Notably, methylated histones can interact with other complexes or adjacent epigenetic modifications to modify their functional outcome on transcription [36]. For example, the combination of H3K4me3 and H3K27me3 poises genes for transcription [40].

### 2.4. Histone Variants and Chromatin Regulators

In addition to modifying tails of existing histones, nucleosome function is further diversified by the presence of histone variants, which can replace canonical histones to change the composition of histone types in the nucleosome [41,42,43] (Figure 2B,C). Histone variants are non-allelic counterparts of canonical histones that typically vary from canonical histones by only a few amino-acids, yet they can influence chromatin in three majors ways: (1) By altering nucleosome stability [44]; (2) By recruiting a unique set of chromatin regulating proteins compared to canonical histones [45,46]; and (3) By being subject to their own histone PTMs that can further influence their function in chromatin [47]. A particularly important property of histone variants in non-dividing cells of the brain is that their transcription is replication independent, which contrasts the replication-dependent transcription of canonical histones that is restricted to DNA replication in somatic cell division [48]. As such, histone variants become the primary source of replacement histones in neurons [41], indicating that their functional relevance in mature neurons is relatively higher compared to non-canonical histones.

Histones in the H3 and H2A families regulate histone-histone and histone-DNA binding, whereas histones in the H2B and H4 families only mediate histone–histone binding [49]. Research on H3 and H2A variants provides examples of the many ways in which histone variants can influence the chromatin landscape. For example, centromeric protein A (CENP-A), a variant of histone H3, is incorporated at centromeres and is fundamental for the assembly of kinetochore and chromosome segregation [50]. Additionally, nucleosomes containing the variant H3.3 are less stable compared to canonical H3 [44] and H3.3 can recruit the nucleosome remodelling deacetylase (NuRD) complex to active promoters [51], linking H3.3 with transcriptional activation. Conversely, the H2A variant H2A.Z is associated with more stable nucleosomes [44,52] and has been implicated in diverse and somewhat contradictory functions, including heterochromatin compaction [53], as well as marking active genes and enhancers [54]. This dual role of H2A.Z is subject of intense investigation, but may be associated with different PTMs on H2A.Z and neighboring histones, or interactions with distinct variants in the same nucleosome [54]. Taken together, histone variants constitute a diverse and dynamic epigenetic mechanism that can have profound effects on transcription and cellular processes.

### 2.5. Histone Variant Chaperones

In nucleosomes, histones can be exchanged for either a canonical histone or a histone variant, in a process termed histone variant exchange (Figure 2B); or they can be removed and subsequently replaced by the same histone type, in a process termed histone turnover [41,42,55] (Figure 2C). Histone chaperones are proteins that contribute to basal incorporation of histones into chromatin and play roles in histone variant exchange and turnover. Histone chaperones can be highly specific, whereby individual chaperones may selectively regulate a single histone type and be selective for either deposition or removal of that histone [56]. This high level of specialization contributes to the positioning of variants in both euchromatin and heterochromatin and the mediation of different downstream functions. For example, H3.3 can be deposited into chromatin at highly transcribed regions by the histone regulator A (HIRA) complex [57,58], but can also be found at telomeres and repetitive heterochromatic regions where it is deposited by a complex comprising alpha thalassemia, mental retardation syndrome X-linked (ATRX), and death domain associated protein (DAXX) [59,60,61]. Notably, histone variant chaperones act in large chromatin remodeling complexes that often carry other proteins involved in transcription. For example, following double strand breaks (DSB), the p400/Tip60 chaperone complex rapidly incorporates H2A.Z at the break site and the histone acetylase Tip60 concomitantly acetylates H4 tails [62,63]. Interestingly, H2A.Z accumulation at the break is only transient and its rapid removal is mediated by the chaperone Anp32e [62,63], suggesting that regulation of histone variants by their chaperones confers a high level of selectivity in functional outcomes attributed to histone variants.

### 2.6. Memory Formation and Maintenance

To understand how epigenetic mechanisms contribute to memory, it is vital to first understand some basic properties of memory formation and maintenance. Memory formation is a protracted process marked by distinct consolidation phases, with different molecular factors playing varying roles in different memory phases [64]. For example, inhibition of transcription and protein synthesis shortly after learning disrupts long-term (≥24 h), but not short-term memories [1]. The effectiveness of these manipulations in disrupting memory is restricted to the first few hours after learning, suggesting that the initial memory consolidation window closes approximately 6h after learning [65,66,67,68]. The primary implication of these data is that molecular events that occur shortly after learning establish behavioral phenotypes that emerge or persist at delayed time points, even if the molecular changes observed during memory consolidation are different than those observed at the delayed time points at which memory is tested. Indeed, hippocampal-dependent learning can induce distinct waves of gene transcription/consolidation [69]. For example, genes that are activated shortly (1 h) after contextual fear learning return to baseline by 24 h, a time point marked by a larger second wave of gene expression [69]. Notably, genes that were differentially expressed at 1 h were primarily immediate early genes, whereas the second wave included genes that encode epigenetic regulators. These findings are consistent with the idea that transcription in the first wave upregulates transcription factors that can initiate a second wave of transcription needed to maintain long-lasting memories [70].

The memory consolidation model is further complicated by reorganization of neural networks that underlie memory storage during recent (within 7 days of learning) and remote (≥7 days after learning) memory phases [71]. Specifically, recent memory recall is initially dependent on the hippocampus, whereas the memory trace is subsequently “downloaded” to the medial prefrontal cortex in the process of systems consolidation [64]. Although most studies tend to focus on molecular events occurring in the hippocampus during recent memory and the cortex during remote memory, there is also evidence that late transcriptional changes in the hippocampus (8–24 h post learning) selectively contribute to the establishment of remote memories [64,72]. As such, studies of epigenetic contributions to memory must be considered in the context of the memory phase that is being studied, particularly in light of growing evidence for stage-specific roles of epigenetic factors in memory [73,74,75].

### 2.7. Role of DNA Methylation in Memory

Some of the earliest evidence for DNA methylation as a regulator of neural plasticity came from studies that reduced methylation levels with pharmacological inhibitors of DNMTs and reported impaired long-term potentiation (LTP) and altered transcription [76]. The in vivo relevance of this effect was demonstrated when DNMT inhibition in the mouse hippocampus impaired fear memory and altered the expression of memory-related genes [18], thus firmly implicating DNA methylation as a key regulator of memory. Notably, this study also showed that changes in DNA methylation are transient, whereby changes in DNA methylation were rapidly induced and reversed within hours of learning, thus contradicting the initial hypothesis that DNA methylation is a stable correlate of memory formation [3,6,18]. However, this transience led to the increasingly accepted view that at minimum, DNA methylation plays a dual role in memory formation—a transient role as a transcriptional regulator during learning and a stable role in remote memory maintenance. Indeed, subsequent studies showed that cortical DNA methylation is stably altered up to 4 weeks after learning [74,77] and that blocking cortical DNA methylation at this time point impairs memory [74]. As such, DNA methylation was shown to have a stage-specific role in memory, whereby it is can be both dynamically and stably altered at different time points and can be maintained across multiple waves of transcription. For example, gene body methylation in hippocampal area CA1 1h after fear learning strongly correlates with methylation at 24 h, suggesting that the hippocampal methylome supports multiple stages of memory formation [69].

Although initial candidate-gene based approaches tended to focus on DNA methylation changes in CpG islands within gene promoters, the advent of genome-wide studies has revealed more extensive methylation changes in distinct regulatory regions. Dnmt3a2 overexpression in neuronal cultures resulted in hypermethylation primarily at intergenic and intronic regions, further supporting the role of DNA methylation beyond promoter methylation [78]. In accordance with this work, Day et al. (2013) demonstrated that in vitro potassium chloride (KCl)-dependent depolarization resulted in hypermethylation at intragenic regions of *Egr1* and *Fos*, two crucial plasticity genes [79]. Indeed, accumulating evidence suggests that DNA methylation regulates gene activity at enhancers [80], exerts spliceosomal control of alternative pre-mRNA splicing [81], and alters the expression of microRNAs [82], suggesting that this epigenetic mark can influence memory through diverse mechanisms.

Overall, a large number of studies now support a role for DNA methylation in memory formation and maintenance, particularly within the hippocampus, prefrontal cortex and the amygdala [13,18,74,79,83,84,85,86,87]. The consensus among these studies is that DNA methylation influences memory by altering transcriptional outcomes, and that the relationship between DNA methylation and transcription varies at different stages of memory formation [69]. The outcomes of these changes are diverse and include altered synaptic activity (LTP and long-term depression (LTD)) [13,88], dendritic morphology and spine density [89], as well as general contributions to synaptic plasticity [90]. However, one factor that has remained elusive is the link between epigenetic changes in the nucleus and the function of engram cells that represent the small sub-population of cells that are directly involved in memory formation [91]. However, one recent study begins to address this issue by implicating DNMT3a in the strengthening of fear memory engrams [78]. Specifically, learning activates a small population of engram cells that undergo structural and transcriptional changes to support memory formation [92], such that artificial reactivation of these cells is sufficient to induce memory recall [93]. However, not all memories and engrams are equally salient and require continuous stabilization of engram ensembles for memories to be accurately and successfully recalled [94]. Recent studies have implicated DNA methylation as a mechanism for stabilizing the engram during consolidation to promote memory strength [78]. Specifically, selective overexpression of Dnmt3a2 in dentate gyrus engram cells (i.e., cells activated during learning) resulted in enhanced fear memory, whereas Dnmt3a2 overexpression had no effect, indicating that DNMT3a2 operates specifically within the context of engram cells to strengthen memories, but cannot direct cells to become part of the engram. Instead, it appears that hypermethylation produced by *Dnmt3a2* overexpression promotes greater stability of the memory circuit, thereby increasing the likelihood of circuit reactivation during memory recall that results in stronger and more efficient retrieval [78]. While this evidence has yet to be directly linked to transcription, it is likely the case that Dnmt3a2 achieves this stabilization by regulating stimulus-dependent transcriptional changes, particularly those involved in synapse fortification and neural plasticity [95]. Indeed, the Dnmt3a2 isoform of DNMT3a is well-suited for activity-dependent transcriptional regulation, as it localizes to euchromatin and acts as a de novo methyltransferase [96].

### 2.8. Hydroxymethylation and Memory

5hmC, the product of TET enzyme activity, may also act as a semi-stable epigenetic mark with a potential role in memory formation [97]. The transient nature of 5hmC is still debated based on evidence that only 1% of all cytosines in the mammalian brain are hydroxymethylated [98], but there is nevertheless evidence for unique regulatory effects of 5hmC compared to 5mC. 5hmC appears to be positively related with transcriptional expression, particularly in gene bodies [97] and this regulatory role may be related to the ability of 5hmC to recruit chromatin regulators, such as MeCP2, which can repress or activate transcription [22,23].

Although a direct role of 5hmC in memory is unclear, several studies have investigated the function of TET proteins, which catalyze the conversion of 5mC to 5hmC [19]. Indeed, accumulating evidence indicates that different TET subtypes play distinct roles in various memory-related processes [83]. Specifically, Tet1 appears to negatively regulate memory, whereby overexpressing its catalytic domain impairs contextual fear memory, increases levels of 5hmC, and upregulates numerous activity-dependent immediate early genes (IEGs) (e.g., *Fos*, *Arc*, *Egr1*, *Homer1*) [83], and *Tet1* depletion enhanced fear memory and object location memory [99]. However, other evidence suggests that Tet1 knockout mice exhibit downregulated expression of multiple neuronal activity-regulated genes (e.g., *Npas4*, *c-Fos*, and *Arc*) and impaired Morris water maze memory [86], indicating a contradictory role in memory. Although the reason for this discrepancy is unclear, it is likely related to developmental effects associated with germline knockout compared to virally-mediated protein depletion in adulthood. As such, these data suggest that the role of Tet1 in memory may be developmentally regulated.

In contrast, the role of Tet2 in memory remains elusive, but there is evidence that it drives the proinflammatory activation of microglia in neurodegenerative diseases, thus making it a potential target for treating neurodegenerative disorders [100]. Tet3 also has a functional impact in the brain, based on recent evidence for a critical role in reward-related memory reconsolidation [101]. Specifically, glutamatergic pyramidal neurons in the dorsal hippocampus require Tet3 for cocaine-associated memory reconsolidation, such that viral vector-mediated Tet3 knockdown impaired reconsolidation and recall in mice [101]. Overall, these data suggest that TET-mediated DNA demethylation influences multiple types of memory by regulating the expression of IEGs and their downstream targets, although additional research is needed to clarify these effects.

### 2.9. Histone Post-Translational Modifications in Memory

Histone PTMs in general, and histone acetylation in particular, have received extensive attention in studies of memory formation over the past 15 years and have been extensively reviewed elsewhere [102], so we will only touch on it briefly. The emerging consensus is that histone modifications are rapidly induced during memory consolidation and return to baseline levels thereafter, thus playing a temporally-restricted role compared to DNA methylation [103]. Histone acetylation was first implicated in synaptic plasticity in *Aplysia*, when it was found that the HAT CREB-binding protein (CBP) and HDAC5 are involved in opposing cellular responses to synaptic activity [104]. This was quickly translated to rodents, where numerous studies show that learning induces histone acetylation [105,106,107,108,109,110,111,112,113,114] and the manipulation of histone acetylation can alter memory. Specifically, genetic or pharmacological manipulation of HDACs/HATs have generally established that increasing histone acetylation enhances memory, whereas decreasing histone acetylation impairs memory [107,109,111,112,113,115,116,117,118,119,120,121,122,123,124,125,126,127,128,129,130,131,132,133,134,135,136,137,138,139,140].

In general, histone modifications appear to regulate transcriptional sensitivity to external stimuli, whereby manipulations that increase histone acetylation preferentially impact activity-induced compared to basal transcription [134]. This link between histone acetylation and permissive transcriptional states may account for the memory-enhancing effects of HDAC inhibitors, which increase learning-induced gene expression and potentiate memory in subthreshold learning paradigms [10,27]. Moreover, this link between histone modifications and “gene inducibility” may explain why genome-wide sequencing studies find weak links between changes in learning-induced histone PTMs and training-induced changes in hippocampal gene expression [77]. It may instead be the case that histone PTMs serve to keep genes in a “ready” or “primed” position so they can rapidly and effectively respond to additional stimuli [10,141], such that induced changes in histone PTMs are in fact establishing the inducible, or poised status of genes for future stimuli.

One implication of PTMs as regulators of poised genes is that PTMs may be especially important for regulating memory strength by promoting gene inducibility to low-threshold learning events. Indeed, artificially increasing histone acetylation with HDAC inhibitors has received tremendous attention for its memory-enhancing properties [142], particularly in mouse models of memory decline (discussed in detail later), further reinforcing the role of histone PTMs as modulators of memory strength. Similarly, drugs targeting enzymes that regulate histone methylation are receiving increasing attention for their potential therapeutic effects [143], making it especially important to understand how these PTMs interact with one another. For example, HDAC inhibition reduces H3K9me2 (a transcriptional repressor) levels in the brain [144], suggesting that these two epigenetic marks may function in concert to alter the chromatin landscape and ultimately, shape memory outcomes.

Until recently, little was known about the metabolic factors required to provide the necessary acetyl-coA for histone acetylation. Acetyl-CoA synthetase 2 (ACSS2) is highly expressed in mouse hippocampal neurons [145] and it has recently emerged as a direct regulator of histone acetylation that gets recruited to transcriptionally active chromatin to supply the acetyl donor, acetyl-CoA [146]. One way in which acetyl-CoA can be generated for histone acetylation is through ACSS2-mediated breakdown of acetate into acetyl-CoA [146,147], such that ACSS2 promotes acetylation at several lysine residues (H3K9ac, H4K5ac, and H4K12ac) and its depletion disrupts IEG expression and impairs memory [146]. These data are beginning to shed light on upstream regulatory processes that influence acetylation levels and demonstrate the regulatory complexity that acts upon the epigenome.

### 2.10. Histone Variants in Memory

Histone variants were recently identified as novel regulators of neural plasticity that have the potential to act as stable epigenetic markers of memory [41,42,55,75,148]. As with DNA methylation and histone PTMs, histone variants are highly sensitive to learning and their dynamic nature is a key feature of memory formation. Specifically, learning results in the rapid removal of histone H2A.Z from promoters of upregulated genes [42,55], suggesting that activity-regulated H2A.Z removal promotes learning-induced gene expression. Similarly, histone H3.3 undergoes extensive activity-induced turnover on promoters and gene bodies of transcriptionally upregulated genes [41], emphasizing the importance of histone variant dynamics in transcriptional regulation during memory consolidation. However, the mechanisms that regulate activity-mediated histone dynamics, particularly for H2A.Z, are still unclear. Studies in non-neuronal cells showed that Anp32e is an H2A.Z-specific histone chaperone that removes (but does not deposit) H2A.Z from chromatin [149,150], but it is not yet known if this chaperone also regulates activity-mediated H2A.Z removal observed during learning. Moreover, H2A.Z regulation is highly complex and includes multiple types of deposition machinery, including the Tip60-p400 complex that is involved in incorporating H2A.Z into chromatin [151]. Tip60 can be pharmacologically manipulated with a Tip60 inhibitor, Nu9056, which reduces H2A.Z binding in neurons and produces memory-stage specific effects on memory [75], thus implicating this factor as a regulator of H2A.Z incorporation in neurons. Similarly, H3.3 binding is regulated by multiple chaperones, although activity-mediated regulation of H3.3 turnover has been attributed primarily to the histone chaperone HIRA [41]. As such, these data suggest that multiple chromatin regulators can influence the removal and incorporation of histone variants, thus providing a mechanism for highly specific histone regulation.

Interestingly, H2A.Z and H3.3 have opposite effects on memory recall, whereby viral vector mediated H2A.Z depletion in the mouse hippocampus enhances memory [42,55] and inhibition of H3.3 turnover impairs memory [41]. Notably, H2A.Z can regulate both recent and remote memories, as evidenced by stable shifts in cortical H2A.Z incorporation 7 and 30 days after fear learning [42,75] and enhancement of remote memory by cortical H2A.Z depletion before training [42]. Moreover, indirect (via Nu9056 injection 1h before recall) inhibition of H2A.Z binding 30 days after training transiently impairs remote memory [75], indicating that H2A.Z may be required for effective recall of remote memory. These data suggest that stable epigenetic changes preferentially occur on genes that are relevant for synaptic function, and that H2A.Z may be similar to DNA methylation in its capacity as a stable marker of memory formation.

The latter is especially interesting because H2A.Z and DNA methylation are mutually antagonistic marks and it is likely that one of these modifications dictates the incorporation of the other [152]. For example, transient H2A.Z depletion during embryonic development in zebrafish leads to DNA hypermethylation [153] and inhibition of DNA methylation in human colon cancer cells increases genome-wide H2A.Z occupancy [154]. Although the interplay between these two modifications has yet to be established in the hippocampus, we did show that genome-wide H2A.Z binding occurs preferentially on CpG islands, which tend to be depleted of DNA methylation [55]. Together, these data suggest that histone variants and DNA methylation may interact to regulate memory, although this relationship needs to be directly studied.

Despite recent progress in our understanding of histone variants, this subfield of neuroepigenetics is in its infancy and many open questions remain. For example, histone variants are subject to the same histone PTMs as canonical histones, but these modifications are not typically taken into consideration in studies of histone variants. There is evidence that H2A.Z is differentially acetylated 24h after fear conditioning and as such, its acetylation may play a role in the ongoing process of systems consolidation [75], but the specific function of H2A.Z acetylation remains to be determined. Moreover, histone variants are typically encoded by multiple genes that may have differential roles in neural plasticity. For example, the H3.3 encoding gene *H3f3b* is preferentially susceptible to activity-induced regulation in neurons compared to *H3f3a* [41], and the H2A.Z encoding genes *H2afz* and *H2afv* regulate distinct transcriptional programs in cultured cortical neurons [155]. Moreover, the role of H2A.Z in memory depends on the type of memory task and the sex of the animal, whereby inducible conditional knockout of both H2A.Z-encoding genes improves fear memory only in males, but improves non-aversive spatial memory in male and female mice [156]. These differences may be due to interactions of H2A.Z with sex hormones, whereby H2A.Z is required for the effects of androgen receptor on fear memory in male mice [157,158]. As such, these data suggest that histone variants in general and H2A.Z in particular, are subject to varying types of upstream regulators that can fine-tune their role in memory formation.

### 2.11. Epigenetic Underpinnings of Memory Impairment in Aging

A notable feature of epigenetic marks is that their levels and genomic occupancy varies across development and as such, changes have been observed both with brain maturation and aging [159,160,161]. Given the dynamic nature of these marks over maturation, it is not clear whether changes in the epigenome reflect normal shifts and adaptations over the life span, or underlie pathological conditions, such as memory impairment. Here, we discuss the emerging evidence for the changing levels and function of epigenetic marks in aging and discuss their relevance to pathology, as well as their potential to serve as therapeutic targets for these afflictions.

### 2.12. Transcriptional Shifts in the Aging Brain

Given the vital role of epigenetics for transcriptional regulation in memory, an extensive effort has been made to characterize epigenetic and transcriptional dysfunction in age-related memory decline. Indeed, aging is associated with altered gene expression in both the human and rodent brain, although the specific nature of the transcriptional shift remains unclear. For example, aged rats (17–22 months) exhibit general up-regulation of gene expression in all three hippocampal sub regions compared to young controls (5–6 months) [162] and in the whole hippocampus in both rats and mice [163,164]. In contrast, others report a preponderance (75%) of downregulated compared to upregulated genes in the hippocampus of aged (15.5 month) compared to young (4 month old) mice [55], or comparable numbers of up- and down-regulated genes in the mouse hippocampus and cortex [165,166]. The reason for discrepancies across studies is not clear and may reflect variable age (ranging from 15 to 22 months), use of different mouse strains, use of mice vs. rats, or memory impairment status. Indeed, 15.5 month old mice may still be cognitively intact [55], whereas 16–18 months-old mice tend to exhibit more consistent memory decline [167,168], suggesting that different studies may be quantifying transcriptional changes that act as precursors to memory decline, whereas others reflect the presence of cognitive decline. It is also entirely possible that some of the changes observed may not be pathological and instead reflect normal transcriptome reorganization with aging, consistent with age-related transcriptional changes observed in the post-mortem brain of cognitively intact human subjects [169]. Specifically, when compared to young (20–59 years) controls, aged subjects (60–99 years) had a large number of differentially expressed genes in the superior frontal gyrus and postcentral gyrus, with the majority of genes being down-regulated. The same study found equal numbers of up- and down-regulated genes in the hippocampus and entorhinal cortex [169], suggesting that variability in brain region under study can also contribute to variable findings in age-related changes in gene expression. Nevertheless, all of these studies do show a change in transcriptional profiles with age and some evidence suggests that downregulated genes in these studies are typically involved in synaptic plasticity, whereas up-regulated genes include immune and inflammatory pathways, cellular organization, and chromatin organization [162,164,166,169,170,171,172].

The recent boom in single-cell transcriptomic studies has helped explain some discrepant transcriptional changes in brain aging by revealing distinct transcriptional profiles across cell types [173,174,175]. For example, single-cell transcriptome analyses comparing whole brain lysates from young (2–3 months) and old (21–22 months) mice find that differently expressed genes in the aged brains fall into three categories: (1) genes that change similarly across cell types; (2) genes that exhibit age-related changes only in in certain cell types; and (3) genes that change bidirectionally across cell types [175]. As such, using mixed cell populations can dilute out difference, or overemphasize one type of change over another, depending on cell types that may be over represented in different tissue types or preparations. Nevertheless, some similarities do emerge between single cell and bulk tissue data, whereby much of the single-cell transcriptomic work emphasizes age-related changes in inflammatory cell types and genes [174,175,176,177,178].

Reductions in the expression of genes that maintain synaptic function may be a common contributing factor to altered memory processing with aging. In addition to baseline changes that occur in aging discussed above, there are also abnormalities in learning-induced gene expression. One study found an especially stark age difference, whereby aged (16 months) mice had a complete lack of learning-induced gene induction in the hippocampus compared to young mice [168]. However, others did not found such dramatic shifts in learning-induced gene expression and instead report more comparable levels of gene induction in aged and young rodents e.g., [55,162,163,166,179]. This discrepancy cannot be completely explained by differences in cognitive performance since studies that compared transcriptional profiles in young and aged animals that were cognitively intact vs. cognitively impaired still show learning-induced gene expression changes, irrespective of impairment [162,163,179]. Notably, aged and young mice appear to respond to learning tasks with widely divergent transcriptional programs [55], suggesting that aging may be associated with activation of distinct gene networks rather than impaired induction of gene expression with aging. Overall, aging is associated with altered transcriptional programs that may be especially relevant for synaptic function and as such, may be linked with memory decline. Given the role of epigenetic factors in regulating transcriptional outcomes, accumulating evidence shows that disorganization of the epigenome may drive altered transcriptional outcomes and memory impairment [180,181,182].

### 2.13. Epigenetic Changes in Aging

It is likely that transcriptional changes in the aged brain are elicited by age-related changes in the epigenome. There is consensus that epigenetic factors, including histone PTMs, histone variants, and DNA methylation are altered in the aged brain [183,184,185,186,187,188]. However, the direction of changes in specific epigenetic marks are highly variable across studies and may reflect differential use of brain regions, learning tasks, and age. One explanation for variable results is that age may not always lead to memory decline. Indeed, several studies find differences in either specific epigenetic marks or a change in the magnitude of the epigenetic signal in aged rodents that are memory impaired, compared to those that are not [108,168,189,190]. Nevertheless, other studies suggest that age-related epigenetic changes are pre-programmed in some way [191], based on evidence for similar epigenetic changes across age, brain regions, and species [41,108,192,193,194,195]. Further, there is evidence for coordinated shifts that may increase susceptibility to memory decline, including accumulation of histone variants and dysregulation of metabolic processes that generate precursors for epigenetic modifications [41,42,196,197,198].

## 3. Histone Post-Translational Modifications

### 3.1. Age-Related Histone Acetylation Changes in the Rodent Brain

Post-translational histone modifications, particularly histone acetylation, have been extensively studied in relation to transcriptional and memory changes in the aging rodent brain. Reports of global and residue-specific acetylation changes reveal complex activity- and brain region-specific changes that differ across studies. For example, one study found that young (6 months) and aged (24 months) rats had comparable basal levels of H3K9ac and bulk H3 acetylation in area cornu ammonis (CA) 1, CA3, and dentate gyrus (DG) and similar learning-induced changes in H3 acetylation in response to training in the Morris water maze [108]. In contrast, H3K9ac decreased in the CA1 and bulk H3 acetylation increased in area CA1, CA3, and DG compared to baseline in young rats [108], suggesting that learning- and age-related changes in hippocampal H3 acetylation may occur with a high level of residue and brain-region specificity. Age differences may be particularly subtle in some instances, whereby spatial learning increased global H3 acetylation both in young (4 months) and aged (18–20 months) mice, but the magnitude of change was greater in aged mice [199], suggesting that more cells may undergo learning-induced epigenetic changes as the brain ages. Some studies report opposite results from one another, whereby hippocampal H3K9ac increased with contextual fear learning in young (3 months) and aged (16 months) mice [168], but was downregulated in young compared to aged mice after Morris water maze learning [108]. One possibility for this discrepancy is the use of whole hippocampus [168] vs. CA1 [108], suggesting that more selective circuit-level changes can be lost when signal from all hippocampal subregions is combined at the level of the whole hippocampus. Consistent with this interpretation, age dependent changes in histone PTMs vary with brain region and the type of memory task in young (4 months) and old (18–20 months) mice [199], indicating that the pattern of age-related epigenetic change is specific for the cellular and signaling context that occurs with different types of tasks and brain regions.

Divergent age-related changes across studies have also been reported for H4 acetylation [108,168,199,200,201], whereby some studies report reduced hippocampal H4 acetylation in aged (20-month-old) compared to young (3-month-old) mice [201], and others report higher H4 acetylation in all hippocampal subfields of aged (24-month-old) rats [108] under baseline conditions. In addition, Morris water maze learning increased H4 acetylation in CA1 but decreased in DG of memory-impaired aged rats compared to their baseline [108], but there are also reports of smaller increases in global H4 acetylation in DG, CA1, and striatum of aged (18–20 months) compared to young mice in response to Morris water maze learning [189]. Taken together, these findings provide evidence that distinct epigenetic changes occur with aging and may underlie the shift from healthy to pathological aging, but that trajectories of epigenetic aging may not be linear, such that distinct types of age-related changes in PTMs can be observed under different conditions.

Overall, rodent studies of acetylation demonstrate shifts in the magnitude of the epigenetic signal under baseline and learning-induced conditions, although there are many discrepancies on the specific type of change observed in different studies. This may partly be due to the use of mixed cell populations, as learning-induced epigenetic changes differ in neurons compared to glia [77], such that prevalence of different cell types or different brain regions may lead to deviations across studies. Moreover, age differences may vary based on the lysine residue under consideration, based on evidence that only certain residues show age-related changes when multiple marks are measured in the same study [168]. Such residue-specific changes may be caused by age-related changes in the activity of specific HATs and HDACS, which can also be dysregulated in the aged rodent brain [202,203,204]. As such, these studies support the idea that the epigenome is dysregulated in the aging brain, but the specific type of PTM alteration cannot be identified as a marker of aging until further controlled research clarifies the extensive sources of variability observed across studies. Alternatively, the lack of a single PTM that is associated with age-related cognitive impairment across studies could instead suggest that aging involves the loss of coordination of epigenetic marks and their writers/readers.

### 3.2. Age-Related Histone Modifications in the Human Brain

As with rodents, imaging and post-mortem studies in human brains have reported age-, sex- and brain-region specific changes in histone acetylation. For example, genome wide sequencing of H4K16ac in the lateral temporal lobe showed that this mark increased at approximately 20,000 loci in aged subjects compared with young controls and was positively correlated with gene expression, suggesting that increased H4K16ac may promote overactive transcription in aging [205]. In contrast to age-related accumulation of H4K16ac on upregulated genes, H3K27ac levels are reduced with age on genes that are differentially expressed in the prefrontal cortex of old subjects when compared to young [206]. The age-related loss of H3K27ac at promoters occurred at both up- and down-regulated genes [206] and as such, may not have a precise relationship with transcription. However, broad H3K27ac loss occurred throughout the gene-bodies of inflammatory genes that were upregulated with age, suggesting the loss of this PTM in gene bodies may promote transcription of distinct gene categories [206]. Consistent with altered histone acetylation levels, age-related changes in HDAC levels have also been reported. Specifically, HDAC positron emission tomography (PET) revealed increases in in vivo HDAC expression in cerebral white matter beginning at 35 years-of-age [193]. Using post-mortem tissue, the same study found selective upregulation of HDAC1 and 2, but not 3 and 6, in white matter of older adults (mean age 85 years) compared to young adults (mean age 18 years), suggesting that age related changes in HDAC levels are driven by HDAC1 and HDAC2 [193]. Notably, learning-induced downregulation of HDAC2 is lost in aged memory impaired rats [108], suggesting that age-related changes in epigenetic regulation is relevant in humans.

### 3.3. Age-Related Changes in Histone Methylation

Other PTMs have not been as extensively examined in the aging brain, though a few studies have observed age-related changes in histone methylation in rodents [190,192,195,202]. For example, whole brain lysates from SAMP8 mice show increased H3K27me, but decreased H3K36me, H3K79me, and H4K20me [190]. Age-related changes in histone methylation also occur in structures important for memory, as bulk H3K4me3 increased in CA regions of rats with age-related memory decline (19–22 months) compared to young (3 months) controls [195]. In addition, only young rats showed bulk increases in H3K4me3 and H3K9me2 in response to novel object recognition training, suggesting that impaired responsiveness in histone methylation may promote memory impairment in aged rats [195]. Notably, both learning and H3K4me3 deficits were reversed with environmental enrichment [195], indicating that histone methylation remains plastic in aged rats and can be modified with environmental intervention. One potential mechanism for altered levels of H3K9me2 in aging may be linked with changes in a long non-coding RNA, *NEAT1*, which regulates H3K9me2 levels via interaction with the methyltransferase euchromatic histone lysine methyltransferase 2 (EHMT2), and accumulates in the aged mouse hippocampus [192]. To our knowledge, age-related changes in histone methylation have not yet been characterized in the human brain. However, H3K4me2 increases over the lifespan in enhancers and promoters of the prefrontal cortex of rhesus macaque, a shift that is positively associated with transcription, suggesting an age-related shift to open chromatin [194]. The conserved changes in H3K4me2 across species suggests that age-related changes in this mark may reflect a general mechanism of senescence, rather than sporadic events.

## 4. Manipulating Histone PTMs to Treat Age-Related Memory Impairment

Given that HDACs are negative regulators of histone acetylation, there has been extensive interest in HDAC inhibitors (HDACi) as therapeutic targets for age-related memory decline. Despite the fairly complex pattern of histone modifications in the aging brain, broad HDAC inhibition typically produces pro-cognitive effects in the aged brain [122,168,172,195,207,208], although some studies have failed to see mnemonic rescue [108,199]. Given the hypothesized role for histone acetylation in priming genes to respond to stimuli (see section on histone post-translational modifications in memory), this beneficial effect may be especially evident under subthreshold learning conditions, whereby HDACi treatment can result in an enhanced transcriptional response to external stimuli [209]. Age-related changes in HAT proteins may also contribute to memory decline. For example, Tip60 supports memory through H4K12ac [113], a mark associated with age-related memory loss [168]. Interestingly, Tip60 can act in a complex with the nuclear adaptor protein Fe65 and the intracellular domain of amyloid precursor protein (APP), which induces transcriptional activation and H4 acetylation [210,211]. Although manipulation of this complex has not been tested for the remediation of age-related cognitive impairment, loss of Tip60 in transgenic Alzheimer’s disease models in *Drosophila* increases APP-mediated apoptosis, an effect that is blocked by Tip60 overexpression [212]. This finding indicates that the Tip60-APP-Fe65 complex regulates pathological changes that underlie cognitive impairment in neurodegenerative disease.

Other therapeutic strategies that boost histone acetylation also show promise for treating age-related cognitive decline. Manipulating the levels of acetyl-CoA that are readily available for histone acetylation modulates gene transcription and hippocampal-dependent memory (as discussed in histone post-translational modifications in memory) [146,147], and has recently been shown to attenuate age-related cognitive impairment [196]. Specifically, levels of acetyl-CoA are reduced in the SAMP8 mouse model of accelerated aging, and pharmacological compounds that elevate acetyl-CoA (via inhibition of acetyl-CoA carboxylase), increase bulk H3K9ac, restore transcriptional shifts associated with aging, and attenuate cognitive deficits [196]. Other proteins involved in the availability of substrates for epigenetic modifications have also been implicated in aging. For example, s-Adenosylmethionine (SAM), the universal methyl donor important for histone and DNA methylation, is altered throughout the body of mouse species with atypical lifespans [197,198,213]. Although SAM has not been explicitly implicated in age-related memory impairment, it is also altered in other neurodegenerative and neurodevelopmental diseases [213]. These data are particularly notable because it suggests that metabolic shifts which affect availability of epigenetic pre-cursors could underlie age-related changes in PTMs and DNA methylation.

Overall, this work implicates histone modifications in cognitive decline associated with advanced chronological age. The discrepancies between global and residue specific changes, as well as basal and learning-related changes in histone acetylation suggest that remediation of age-related memory decline will require more selective epigenetic targeting than is possible with HDAC inhibitors or other drugs that modulate pan acetylation.

## 5. Aging and Histone Variants

**Figure 3:**
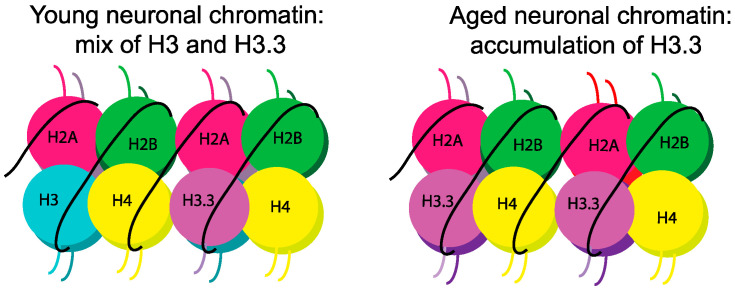
Histone variant H3.3 accumulates during aging. H3.3 is present at low levels in young neuronal chromatin, in which total H3 is represented by a mix of H3.3 and canonical H3 (**left**). During aging, H3.3 levels increase, and the variant becomes the predominant H3 histone in chromatin (**right**).

Recently, histone variants were identified as additional epigenetic regulators of aging that offer alternative therapeutic targets to HDAC inhibitors. One downside of HDACi is their wide-spread effect on acetylation across histone and non-histone proteins, and across various histone types and genomic loci [214]. Histone variants are of particular interest for aging because they are structurally distinct proteins that can, in theory, be directly manipulated without the need to target less specific regulatory enzymes (e.g., HDACs), thus providing more specific therapeutic targets. Unlike canonical histones, histone variants are replication-independent, and as such, form the primary source of replacement histones in post-mitotic neurons [41,215]. This provides a mechanism by which histone variants may accumulate with age, as has been demonstrated robustly for H3.3 [41]. In the embryonic brain, H3.3 represents only a small proportion of total H3, but it becomes the dominant form of H3 in the adult human and mouse brain (Figure 3) [41]. These data indicate that normal histone turnover results in preferential incorporation of histone variants at the expense of replication-coupled canonical histones in the brain.

Notably, the extensive accumulation of H3.3 likely occurs because H3.3 is the only replication independent H3 variant, whereas H2A variants are much more numerous, suggesting that each H2A histone is less likely to accumulate to such an extreme extent [43]. Our group recently reported that the H2A variant histone H2A.Z accumulates in the hippocampus of aged (15-months-of-age) compared to young adult (4-months-of-age) mice [55], suggesting that H2A variants can also increase in aged neurons. This is especially relevant for age-related memory decline because several lines of evidence indicate that H2A.Z is a memory suppressor [42,55,75,156], such that its accumulation may promote age-related memory decline. Interestingly, mice with H2A.Z accumulation were tested at 15.5 months, an age that typically precedes memory decline, and as such, H2A.Z accumulation in this study may serve as a precursor to the emergence of cognitive deficits.

**Figure 4:**
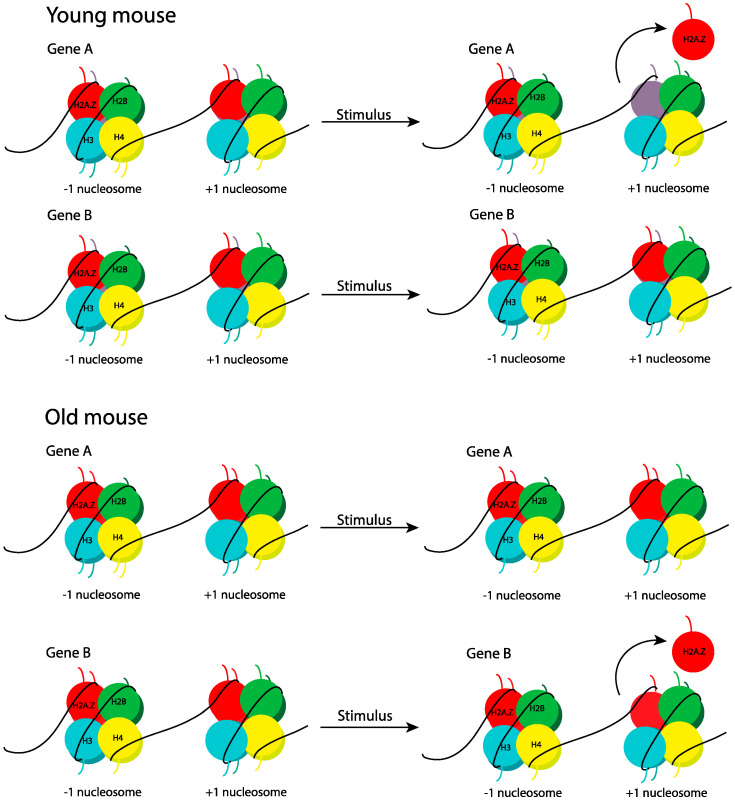
H2A.Z displays a different pattern of learning-dependent removal in young and aged mice. Histone variant H2A.Z is abundant at the −1 (upstream from transcription start site) and +1 (downstream from transcription start site) nucleosomes of several active genes in neuronal chromatin in young and aged mice. Following a learning event, H2A.Z is removed from the nucleosome of numerous genes (**top**), but the removal occurs for different genes in young (**top**) and aged (**bottom**) mice.

A key feature of histone variant function in memory is their dynamic regulation in response to learning (see section on Histone variants in memory) [41,42,55]. When comparing H2A.Z dynamics in aged and young mice, both age groups showed a loss of H2A.Z from upregulated genes in response to learning, but the degree of removal was lower in aged compared to young mice (Figure 4) [55]. In addition, H2A.Z was removed from distinct sets of genes at each age [55], indicating that H2A.Z sensitivity to stimuli shifts to a unique gene network with aging. These findings suggest that dysregulation of basal and learning-related changes in H2A.Z dynamics may contribute to age-related cognitive impairment. This is particularly notable because contextual fear memory deficits typically emerge at 16 to 18-months-of-age when weak training protocols are used [167,168], so epigenetic changes that occur around this time or prior to the onset of cognitive impairment may be causally relevant for subsequent impairment. Indeed, aged mice that showed a dysregulated H2A.Z binding pattern did not yet demonstrate deficits in a robust fear memory protocol [55], but they were impaired in subthreshold training protocol (unpublished observation), suggesting that H2A.Z accumulation may precipitate age-related memory decline. Alternatively, H2A.Z accumulation may be a normal or protective part of aging, as there is evidence that H2A.Z function shifts during early development. Specifically, brain-specific embryonic H2A.Z deletion is detrimental to memory [216], whereas adult H2A.Z depletion is beneficial [42,55,75,156], indicating that roles of epigenetic factors may be affected by the developmental context. In all, these findings suggest that H2A.Z is a potentially more precise therapeutic target for age-related memory decline compared to epigenetic marks that are much more broadly distributed.

## 6. Age-Related Changes in DNA Methylation

As with histone PTMs and variants, DNA methylation is also altered in the aging brain [217,218,219,220]. For example, under basal conditions, DNA methylation in the hippocampus increased at some loci and decreased on other loci in aged (24-months-of-age) compared to young mice (3-months-of-age) [217], suggesting that changes in DNA methylation are not uniform across the genome. Age-related methylation changes were also described in CpG islands of eight of 50 candidate genes examined in the human temporal neocortex [220], reinforcing the observation that site-specific DNA methylation changes are relevant in humans.

A handful of studies have implicated altered DNA methylation in age-related memory impairment. One such study demonstrated that impaired performance on a set-shifting task in aged rats (17 to 22-months-of-age) was associated with redistribution of DNA methylation across the genome in the prefrontal cortex in comparison to young rats (5 to 6-months-of-age) [221]. Specifically, aged rats had high levels of 5mC in the bodies of genes involved in synaptic plasticity (e.g., postsynaptic density, dendrites, the axon terminus, and Ca^2+^ channels) that correlated with decreased expression of these genes [221]. Similarly, candidate-gene studies in the hippocampus found complex age- and learning-related methylation changes at two loci on the *Arc* gene [219]. In CA1, aged rats (24–32 months) had increased methylation at promoter and intragenic regions of *Arc* compared to adults (9–12 months) under baseline conditions, but both age groups effectively demethylated the *Arc* promoter after spatial exploration in the Morris water maze, indicating that dynamic regulation of DNA methylation was retained. Notably, intragenic *Arc* methylation was reduced with learning in aged rates and increased with aging in younger rats [219], consistent with locus-specific dysregulation of DNA methylation observed in genome-wide sequencing studies [221]. Age-related changes in DNA methylation also vary with hippocampal subregion, as promoter methylation of *Arc* in DG was not affected at baseline, but methylation decreased age at the intragenic *Arc* locus decreased with age [219]. Learning resulted in increased methylation at the *Arc* promoter in adult and aged rats, whereas intragenic methylation increased in aged and decreased in adult rats. Similar site-specific changes were reported for *Egr1* methylation in relation to impaired MWM memory in aged (24–32 month-old) rats [218], indicating that site-specific changes in methylation are a key aspect of aging.

How such changes are established is not clear, but aging has also been associated with decreased levels of DNMTs, which are vital for establishing DNA methylation and as such, their dysregulation may underlie altered methylation patterns observed with aging [204,222]. For example, DNMT1 and DNMT3a expression in the cortex and hippocampus is reduced in adult (30 weeks) and aged (80 weeks) compared to young mice (10 weeks) [204]. Likewise, aged mice (18 months) had reduced hippocampal *Dnmt3a2* mRNA expression compared to young mice (3 months) [222]. This decrease was associated with deficits in contextual fear and object recognition memory, both of which were reversed with *Dnmt3a2* overexpression [222], suggesting that loss of Dnmt3a2 is functionally relevant for age-related memory loss. In addition to DNMTs, whose role is to establish DNA methylation, aged mice (18 months) also show a loss of TET2, an enzyme implicated in DNA de-methylation [223]. Reduction in TET2 levels resulted in reduced 5hmC levels, impaired neurogenesis and hippocampal-dependent memory in aged compared to young mice (3 months). As with DNMT3a2, these deficits were restored with *TET2* overexpression [223], indicating that reduced levels of methyltransferase and demethylation-related enzymes are reflective of broader dysregulation of methylation machinery that may culminate in site-specific changes in methylation patterns described above.

## 7. Conclusions

An open question on age-related epigenetic changes is what is driving them. Some studies discussed above suggest that environmental enrichment can reverse age-related changes in histone PTMs and attenuate age-related memory decline [166,195,201,224], indicating that at least some of the age-related changes observed in rodents may be the result of relatively impoverished housing conditions. Additionally, some of the diversity in specific age-related changes across tasks and brain regions may be related to differences in upstream signaling factors that ultimately induce these epigenetic changes, but information on these factors is currently limited. For example, some evidence suggests that histone phosphorylation is induced by ERK/MAPK [225], such that age-related changes in upstream regulatory pathways [166,195,201,224] may also alter epigenetic modifications. Nevertheless, some epigenetic changes do appear to be conserved across species, indicating that at least some age-related shifts may be caused by a shared mechanism related to cellular senescence [194,195].

Data on the accumulation of histone variants suggest that epigenetic changes may be an intrinsic property of aging neurons, as their post-mitotic status restricts their access to canonical histone types with age, thus limiting the available histone pool to replication-independent variants that change the chromatin environment. It is also important to consider the fact that changes in a single chromatin mark may be sufficient to extensively alter the surrounding chromatin. For example, histone acetylation and DNA methylation can influence each other during learning [226] and as such, changes in DNA methylation may be linked with changes in histone acetylation and vice versa. In addition, studies in non-neuronal tissues suggest that H2A.Z tends to be excluded from methylated DNA and vice versa [152] and is found in nucleosomes with more H3 and H4 acetylation [227], suggesting that these factors may influence one another. Indeed, the loss of coordination of epigenetic marks and machinery with age has been suggested to underlie cognitive impairment [108].

Conversely, some of the observed changes may be sporadic and simply reflect epigenetic drift, defined as the gradual alteration of epigenetic patterns over the lifespan due to various exogenous (environmental) and endogenous (stochastic) influences [228,229]. In human post-mortem brain samples, 30% of CpG sites examined had significant change in variability (heteroscedasticity) across age in both the cerebral cortex and cerebellum, with ~90% of changes reflecting an increase in variance [229]. Furthermore, analysis of probes that were commonly represented by both the cortex and cerebellum [229] indicated an increased cortex-cerebellum similarity in the aged cohort, in comparison to the lower ages, indicating that cells across brain regions become more similar as they age, and as such, may reflect a shared neuronal aging mechanism.

Although we have made progress in understanding how the epigenome is altered in the aging brain, a complete understanding of this process will depend on comprehensive investigation of multiple chromatin factors and their integration with changes in upstream regulators.
